# Improving psychological well-being and neurocognitive efficiency in aging: Efficacy of a neurofeedback-guided mindfulness protocol

**DOI:** 10.1192/j.eurpsy.2021.1240

**Published:** 2021-08-13

**Authors:** D. Crivelli, M. Balconi

**Affiliations:** 1 International Research Center In Cognitive Applied Neuroscience – Irccan, Research Unit In Affective And Social Neuroscience, Department Of Psychology, Catholic University of the Sacred Heart, Milan, Italy; 2 International Research Center For Cognitive Applied Neuroscience - Irccan, Research Unit In Affective And Social Neuroscience, Department Of Psychology, Catholic University of the Sacred Heart, Milan, Italy, Milan, Italy

**Keywords:** Wearable neurofeedback, Aging, Neurocognitive efficiency, Mood

## Abstract

**Introduction:**

Mounting evidence shows that mental training activities aimed at increasing self-awareness and self-regulation can better psychophysical wellbeing, as well as on neurocognitive efficiency. Nevertheless, the commitment required by traditional approaches to such activities often discourages their implementation as prevention tools with elderly users. Integrating traditional interventions and new technologies might help to achieve this goal.

**Objectives:**

The study aims at evaluating the effects on cognitive control and self-regulation of a mindfulness protocol supported by a wearable neurofeedback device, comparing young-adults and elderly people.

**Methods:**

Participants completed a three-week experimental (EXP) or control (CONT) training protocol, with daily sessions of practice. The EXP protocol was based on breathing awareness practices executed with the support of the wearable neurofeedback device. In the CONT protocol, participants completed breathing practices while listening to ambient sounds with no feedback. Stress, anxiety and mood levels, cognitive skills, and physiological markers (EEG and autonomic indices) of neurocognitive efficiency and stress were assessed pre-/post-training.

**Results:**

Both young and elderly participants completing the experimental protocol showed a post-training improvement in executive control, a reduction in perceived stress levels, and an improvement of psychophysiological markers of stress regulation. In addition, young participants presented an improvement of EEG markers of attention regulation, while elderly participants showed an improvement of EEG markers of affective regulation and a reduction of subclinical depressive symptoms.

**Conclusions:**

Findings highlights the potential of integrating traditional interventions and new technologies in order to promote subjective well-being and neurocognitive enhancement, especially with elderly users.

